# Gastrointestinal cancer and bilateral hydronephrosis resulted in a high risk of ureteral stent failure

**DOI:** 10.1186/s12894-018-0346-3

**Published:** 2018-05-08

**Authors:** Mari Ohtaka, Takashi Kawahara, Daiji Takamoto, Taku Mochizuki, Yusuke Hattori, Jun-ichi Teranishi, Kazuhide Makiyama, Yasuhide Miyoshi, Yasushi Yumura, Masahiro Yao, Hiroji Uemura

**Affiliations:** 1 Departments of Urology and Renal Transplantation, Yokohama City University Medical Center, 4-57 Urafune-cho, Minami-ku, Yokohama, Kanagawa 2320024 Japan; 2 Department of Urology, Yokohama City University Graduate School of Medicine, Yokohama, Japan

**Keywords:** Ureteral stenting, Malignant ureteral obstruction

## Abstract

**Background:**

Urologists frequently encounter malignant ureteral obstruction (MUO) caused by advanced urological or non-urological malignant disease, but the treatment policy is unclear. The present study examined the risk factors for predicting ureteral stent failure in patients with MUO after ureteral stent insertion and the change in the renal function after retrograde ureteral stent insertion in cases of bilateral hydronephrosis.

**Methods:**

A total of 39 patients who required ureteral stent placement for MUO at Yokohama City University Medical Center (Yokohama, Japan) between February 2007 and May 2016 were included in this study. The age, gender, type of cancer, hydronephrosis side, pre-stenting estimated glomerular filtration rate (eGFR), and eGFR increase were assessed as predictive factors for stent failure. Among these 39 patients, 25 showed bilateral hydronephrosis. Thirteen of these patients had bilateral ureteral stents placed, and the remaining 12 had a unilateral ureteral stent placed. The renal function and overall survival (OS) were analyzed between these two groups.

**Results:**

Among all 39 patients, 9 (23.1%) had stent failure. A univariate analysis revealed that causative disease (gastrointestinal cancer vs. others; *p* = 0.045) and laterality of hydronephrosis (bilateral vs. unilateral; *p* = 0.05) were associated with stent failure. A multivariate analysis revealed that only age (hazard ratio, 0.938; 95% confidence interval, 0.883–0.996; *p* = 0.038) was associated with stent failure. A Kaplan-Meier analysis and log-rank test indicated that having a unilateral ureteral stent placed was not correlated with a lower OS rate than having bilateral ureteral stents placed (*p* = 0.563). Among patients with bilateral hydronephrosis, the increase in the eGFR of those who had bilateral ureteral stents placed was not significantly different from that of those who had a unilateral ureteral stent placed (*p* = 0.152).

**Conclusions:**

We revealed that age > 60 years was helpful for predicting stent failure. MUO due to gastrointestinal cancer and bilateral hydronephrosis may be predictive of stent failure. These factors may help urologists decide the optimal time to perform early percutaneous nephrostomy. These findings suggest that patients with bilateral hydronephrosis do not necessarily need to have a ureteral stent placed into both sides of the hydronephrosis.

## Background

Urologists frequently encounter malignant ureteral obstruction (MUO) caused by advanced urological or non-urological malignancies. The causes of MUO are varied and include primary tumors, metastatic lymph nodes, peritoneal dissemination, and local infiltration. If untreated, progressive obstruction can result in uremia, electrolyte imbalance, urinary tract infections, and low back pain. Effective management must be attempted, but the treatment policy is unclear [1–5]. In patients with MUO, the current management options are retrograde ureteral stent (RUS) placement or percutaneous nephrostomy (PCN) under local anesthesia. RUS is usually considered as the first treatment choice because of its low rate of complications, low invasiveness, and low exchange frequency. However, the stent failure rate is high, with a mean failure rate of 12.2–34.6% [6–11]. Therefore, PCN should be used instead of RUS as the primary procedure in patients who would otherwise be at high risk of stent failure.

Previous studies have identified several factors of stent failure in patients with MUO, including the pre-stenting serum creatinine (S-Cr) level, performance states (PS), and degree of hydronephrosis [12, 13]. We can reduce the number of unnecessary procedures by carefully considering these risk factors. Sang Hoon Song et al. showed that patients with bilateral MUO, especially those ≥55 years of age or with diabetes or a poor baseline renal function, should be considered for early PCN conversion in the dominant functional kidney or in both to preserve the renal function, but few studies have explored the management of bilateral MUO [14].

The present study retrospectively reviewed our institution’s experience with treating MUO using RUS and analyzed the factors predicting stent failure and the prognosis. We measured the pre- and post-baseline eGFR and analyzed the correlation between the increase in eGFR and stent failure in bilateral MUO. We also examined the risk factors predicting ureteral stent failure in MUO patients whose renal function changed after retrograde ureteral stent placement in bilateral hydronephrosis.

## Methods

### Patients

A total of 39 patients who required ureteral stent placement for MUO at Yokohama City University Medical Center (Yokohama, Japan) between February 2007 and May 2016 were retrospectively analyzed in this study. Primary indwelling ureteral stent placement was indicated for a variety of reasons, including pain control of hydronephrosis and improvement of the renal function, as well as for chemotherapy. The indication for RUS or PCN was left to the surgeon’s decision. At our institute, PCN is suggested for patients with non-obstructive hydronephrosis, such as those with direct tumor invasion. In most cases, ureteral stenosis was observed between the upper and uretero-vesicle junction. At our institution, all MUO patients underwent RUS with a rigid cystoscope under local anesthesia under fluoroscopic guidance. In some male patients, we add sacral anesthesia. A 6-Fr 26-cm ureteric stent (Polaris™ Ultra; Boston Scientific, Natick, MA, USA) with a 0.035-mm SENSOR guide wire (Boston Scientific, Natick, MA, USA) was used, and the stent was changed every 3 months. At our facility, we started to use the RESONANCE metallic stent from 2016. Therefore, this study does not include any metallic stents. If RUS failed, the patients were referred for placement of a unilateral PCN tube. We defined stent failure as having to change the ureteral stent before the scheduled ureteral stent exchange time or having to perform PCN. A decreased renal function was defined as an increase in the serum creatinine level. Stent failure also included cases in which a ureteric stent could not be placed initially.

Patients’ age, gender, type of cancer, hydronephrosis side, pre-stenting estimated glomerular filtration rate (eGFR), and eGFR increase were assessed as predictive factors for stent failure. The eGFR increase was defined as the difference between the pre-stenting eGFR and the best post-stenting eGFR. We also analyzed the relationship between stent failure and the overall survival (OS). In addition, from the total population, we extracted the 25 cases of bilateral hydronephrosis and compared the eGFR increase and OS in the 13 patients who received bilateral ureteral stent placement and the 12 patients who received unilateral stent placement. We usually checked the serum creatinine level and CT-KUB every 3 months. Institutional Review Board of Yokohama City University Hospital approved this study and required no written informed consent for all patients due to the retrospective observational study.

### Statistical analyses

Univariate and multivariate logistic regression analyses were performed to determine the predictors of stent failure. Odds ratios (ORs) were computed along with 95% confidence intervals (CIs). The survival duration was defined as the time between the date of RUS and death. A log-rank test was performed for comparisons between stent failure and non-failure groups. *P* values of < 0.05 were considered to indicate statistical significance. The eGFR increase in patients with bilateral hydronephrosis was analyzed by the Mann-Whitney U and chi-squared tests. All statistical analyses were performed using the EZR software program (Saitama, Japan).

## Results

### Patients’ characteristics

The 39 patients included 18 males and 21 females. The median age (range) was 70.0 (40–89) years, and the median observation period (range) was 141 (5–1729) days. The characteristics of the patients, including the pre-stenting s-Cr and eGFR, eGFR increase, laterality of hydronephrosis, and causative disease, are summarized in Table 1. During the observation periods, 9 (23.1%) patients had stent failure and received placement of a unilateral PCN tube, depending on their general condition. Among the patients with colorectal cancer, three had rectal cancer, two had sigmoid cancer, and one had ascending colon cancer. The remaining cases of gastrointestinal cancer were gastric cancer. No significant differences were observed in the stent failure rate among the types of gastrointestinal cancer.

**Table 1:** Patients’ clinical characteristics

Variables	n (%)
Age (median; years)	70.0 (40–89)
Gender	
Male	18 (46.2%)
Female	21 (53.8%)
Observation periods (median; days)	141 (5–1729)
Serum creatinine (median; mg/dL)	3.13 (0.81–19.21)
eGFR (median; ml/min/1.73 m2)	15.6 (1.6–62.2)
eGFR increase rate (median; %)	31.9 (−53.8–2606.2)
Laterality of Hydronephrosis	
Left	5 (12.8%)
Right	9 (23.1%)
Bilateral	25 (64.1%)
Cause disease	
Gastrointenstinal cancer	24 (61.5%)
Gynecological cancer	11 (28.2%)
Lung cancer	1 (2.6%)
Prostate cancer	1 (2.6%)
Unknown primary cancer	2 (5.1%)
Stent failure	
Yes	9 (23.1%)
No	30 (76.9%)

### Stent failure analyses

**Fig. 1:**
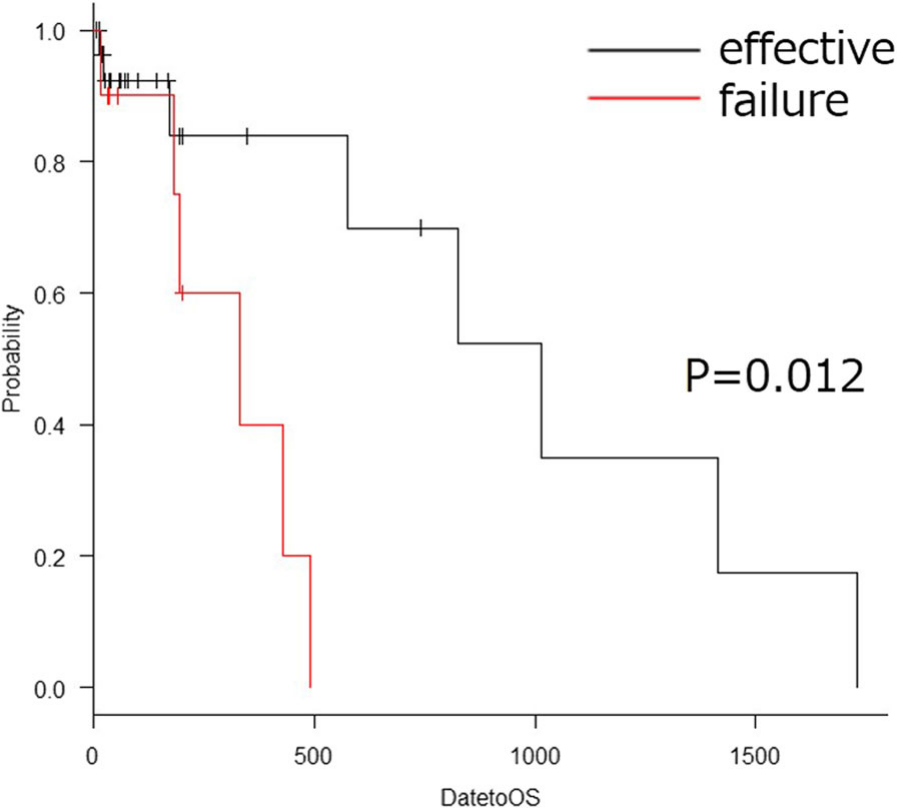
Kaplan-Meier curve for the overall survival according to stent failure

A univariate analysis revealed that the causative disease (gastrointestinal cancer vs. others; *p* = 0.045) and laterality of hydronephrosis (bilateral vs. unilateral; *p* = 0.05) were associated with stent failure, whereas age, gender, eGFR, and eGFR increase were not associated with the stent failure-free survival (Table 2). A multivariate analysis revealed that only age (hazard ratio [HR], 0.938; 95% CI, 0.883–0.996; *p* = 0.038) was associated with stent failure (Table 2). A Kaplan-Meier analysis and log-rank test indicated that stent failure was correlated with a lower OS rate than non-stent failure (*p* = 0.012; Fig. 1).

**Table 2:** Univariate and multivariate analysis for stent failure

	Univariate	Multivariate			
Variables	p value	p value	HR	95%CI	
				Lower	Upper
Age (≥60 years vs < 60)	0.615	0.038	0.938	0.883	0.996
Gender (Male vs Female)	0.178	0.750	1.304	0.253	6.697
eGFR (≤15.6 vs 15.6≥)	0.5	0.761	0.798	0.187	3.407
eGFR increase rate (≤31.9 vs ≥31.9)	0.916	0.813	0.812	0.144	4.562
Cause disease (GI cancer vs others)	0.045	0.201	3.769	0.493	28.81
Laterality of Hydronephrosis (Bi vs Uni)	0.05	0.068	0.117	0.011	1.18

### Bilateral hydronephrosis analyses

**Fig. 2:**
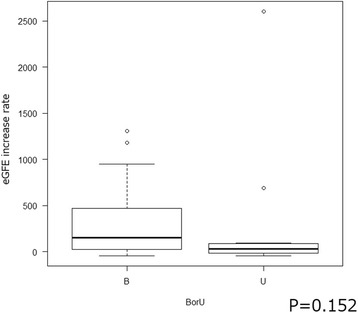
A comparison of the eGFR increase (B: bilateral, U: unilateral)

**Fig. 3:**
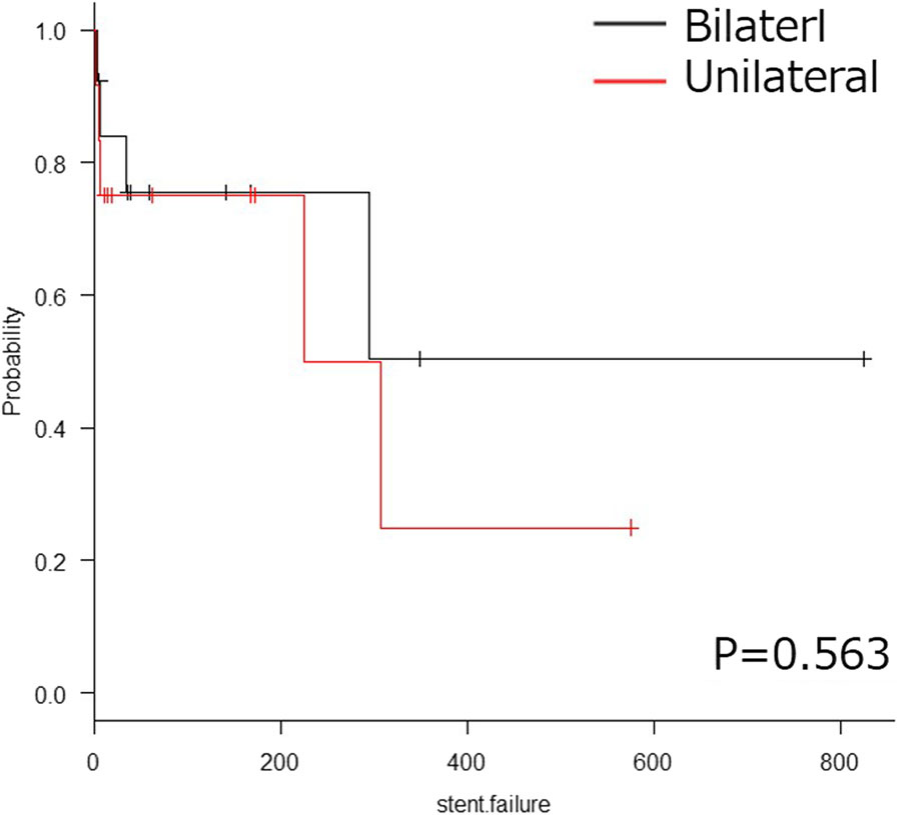
Kaplan-Meier curve for the overall survival according to bilateral or unilateral stenting

The eGFR increase was not significantly different between the bilateral hydronephrosis patients who underwent bilateral stenting and those that underwent unilateral stenting (*p* = 0.152; Fig. 2). In addition, a Kaplan-Meier analysis and log-rank test indicated that unilateral stenting was not correlated with a lower OS rate than bilateral stenting (*p* = 0.563; Fig. 3).

## Discussion

MUO is a frequent complication of advanced hard-to-treat malignancy and indicates a poor prognosis. However, there is no consensus on the appropriate management of MUO, as these patients’ backgrounds vary widely with respect to complications, general condition, the prognosis, and quality of life issues [7, 9, 14]. MUO from malignancy may be because of compression by the primary or metastatic tumor, lymphadenopathy, or tumor direct invasion. Therefore, renal failure and associated symptoms can be improved and maintained by early optimum urinary diversion in some cases.

RUS is common in clinical practice and chosen more often than PCN when attempting to ensure the life expectancy of patients with advanced malignancies [6, 8]. However, the incidence of stent failure is high, possibly due to the high extrinsic pressure on the plastic ureteral stent or invasion of the ureter by tumors, which may lead to the stent’s loss of function [15].

The present study included patients with a good general condition who had not yet received chemotherapy; this may have resulted in a relatively low stent failure rate. Furthermore, Wang et al. said that stent failure was influenced by the anesthesia used [5]. In China, the procedure is usually performed under local anesthesia in an outpatient operating room, which can lead to anxiety and pain during surgery. In Japan, most urologists perform RUS under local anesthesia in an operating room or treatment room. However, no studies have yet explored the association between stent failure and anesthesia, so the relationship remains controversial.

Some studies have reported the risk factors for stent failure. For example, Yu et al. found that middle or lower ureteral obstruction, PS ≥1, and s-Cr before ureteral stent insertion > 1.2 mg/dL were unfavorable predictors of the stent failure-free survival [16]. These factors may help urologists predict the survival time. Kamiyama et al. also showed that primary GI cancer, severe preoperative hydronephrosis, peritonitis carcinomatosa, and a poor preoperative PS were factors influencing stent failure [17]. As these risk factors are still being discussed, there is no consensus on predicting stent failure in MUO. In the present study, the age (> 60 years), causative disease (gastrointestinal disease), and the presence of bilateral hydronephrosis were suggested to be associated with stent failure, whereas gender, pre-stenting eGFR (< 15.6 ml/min/1.73 m^2^), and eGFR increase (< 31.9%) were not associated with stent failure. Some authors in Japan have reported that gastrointestinal disease is associated with a poorer prognosis than other types of malignancy and is reported as a risk factor of stent failure [4.5]. However, few studies have been conducted with the same parameters as those in other countries. We also examined the eGFR, not s-Cr, because of its precision in evaluating the renal function. Patients who undergo RUS for chemotherapy do not necessarily have severe renal failure, leading to a relatively low eGFR increase. This may be why eGFR was not associated with stent failure.

Song et al. suggested that patients with bilateral MUO, especially those ≥55 years of age or with diabetes or a poor baseline renal function, should be considered for early PCN conversion in the dominant functional kidney or in both to preserve the renal function [18]. However, the relative lack of studies has prevented obtaining a consensus about bilateral MUO. In the present study, we examined the eGFR increase in RUS for bilateral hydronephrosis. The increase in the eGFR and OS were not significantly different between those who had bilateral ureteral stents placed and those who had a unilateral ureteral stent placed. This suggests that bilateral stenting may not necessarily be required for bilateral MUO. PCN can be considered the first treatment approach in patients with MUO who are at risk of failure if RUS is performed. These findings suggest that patients with bilateral hydronephrosis do not necessarily need to have a ureteral stent placed into both sides of the hydronephrosis.

Several limitations associated with the present study warrant mention. First, the cases were retrospectively enrolled from only one facility, so there were few cases in our analysis. Second, the patients in whom RUS was attempted but failed because of severe obstruction were included as stent failure cases, which may have led to an increased stent failure rate. Third, we did not analyze the association between stent failure and cancer treatment, like chemotherapy or radiotherapy. Fourth, in the bilateral hydronephrosis analysis, the urologists did not share a common treatment principle concerning stenting style (bilateral or unilateral). Finally, we did not assess the split renal function. It is difficult to assess the renal function routinely in patients with MUO due to their severe performance status.

## Conclusion

This study revealed that age > 60 years, MUO due to gastrointestinal cancer, and bilateral hydronephrosis may be predictive of stent failure. These factors may help urologists decide on a treatment approach.

## Abbreviations

MUO: Malignant ureteral obstruction
PCN: Percutaneous nephrostomy
RUS: Retrograde ureteral stent

## References

[1] Abt D, Warzinek E, Schmid HP, Haile SR, Engeler DS (2015). Influence of patient education on morbidity caused by ureteral stents. Int J Urol.

[2] Elsamra SE, Leavitt DA, Motato HA, Friedlander JI, Siev M, Keheila M, Hoenig DM, Smith AD, Okeke Z (2015). Stenting for malignant ureteral obstruction: tandem, metal or metal-mesh stents. Int J Urol.

[3] Rosenberg BH, Bianco FJ, Wood DP, Triest JA (2005). Stent-change therapy in advanced malignancies with ureteral obstruction. J Endourol.

[4] Yossepowitch O, Lifshitz DA, Dekel Y, Gross M, Keidar DM, Neuman M, Livne PM, Baniel J (2001). Predicting the success of retrograde stenting for managing ureteral obstruction. J Urol.

[5] Wang JY, Zhang HL, Zhu Y, Qin XJ, Dai BO, Ye DW (2016). Predicting the failure of retrograde ureteral stent insertion for managing malignant ureteral obstruction in outpatients. Oncol Lett.

[6] Izumi K, Mizokami A, Maeda Y, Koh E, Namiki M (2011). Current outcome of patients with ureteral stents for the management of malignant ureteral obstruction. J Urol.

[7] Jeong IG, Han KS, Joung JY, Seo HK, Chung J (2007). The outcome with ureteric stents for managing non-urological malignant ureteric obstruction. BJU Int.

[8] Wenzler DL, Kim SP, Rosevear HM, Faerber GJ, Roberts WW, Wolf JS (2008). Success of ureteral stents for intrinsic ureteral obstruction. J Endourol.

[9] Kouba E, Wallen EM, Pruthi RS (2008). Management of ureteral obstruction due to advanced malignancy: optimizing therapeutic and palliative outcomes. J Urol.

[10] Rosevear HM, Kim SP, Wenzler DL, Faerber GJ, Roberts WW, Wolf JS (2007). Retrograde ureteral stents for extrinsic ureteral obstruction: nine years' experience at University of Michigan. Urology.

[11] Kanou T, Fujiyama C, Nishimura K, Tokuda Y, Uozumi J, Masaki Z (2007). Management of extrinsic malignant ureteral obstruction with urinary diversion. Int J Urol.

[12] Chung SY, Stein RJ, Landsittel D, Davies BJ, Cuellar DC, Hrebinko RL, Tarin T, Averch TD (2004). 15-year experience with the management of extrinsic ureteral obstruction with indwelling ureteral stents. J Urol.

[13] McCullough TC, May NR, Metro MJ, Ginsberg PC, Jaffe JS, Harkaway RC (2008). Serum creatinine predicts success in retrograde ureteral stent placement in patients with pelvic malignancies. Urology.

[14] Modi AP, Ritch CR, Arend D, Walsh RM, Ordonez M, Landman J, Gupta M, Knudsen BE (2010). Multicenter experience with metallic ureteral stents for malignant and chronic benign ureteral obstruction. J Endourol.

[15] Goldsmith ZG, Wang AJ, Banez LL, Lipkin ME, Ferrandino MN, Preminger GM, Inman BA (2012). Outcomes of metallic stents for malignant ureteral obstruction. J Urol.

[16] Yu SH, Ryu JG, Jeong SH, Hwang EC, Jang WS, Hwang IS, Yu HS, Kim SO, Jung SI, Kang TW (2013). Predicting factors for stent failure-free survival in patients with a malignant ureteral obstruction managed with ureteral stents. Korean J Urol.

[17] Kamiyama Y, Matsuura S, Kato M, Abe Y, Takyu S, Yoshikawa K, Arai Y (2011). Stent failure in the management of malignant extrinsic ureteral obstruction: risk factors. Int J Urol.

[18] Song SH, Pak S, Jeong IG, Kim KS, Park HK, Kim CS, Ahn H, Hong B (2015). Outcomes of stent-change therapy for bilateral malignancy-related ureteral obstruction. Int Urol Nephrol.

